# Effect of curcumin on inflammatory biomarkers and iron profile in patients with premenstrual syndrome and dysmenorrhea: A randomized controlled trial

**DOI:** 10.14814/phy2.15763

**Published:** 2023-07-02

**Authors:** Amir Talebpour, Mahtab Mohammadifard, Reza Zare Feyzabadi, Sara Mahmoudzadeh, Hadis Rezapour, Mansoore Saharkhiz, Mahboube Tajik, Gordon A. Ferns, Afsane Bahrami

**Affiliations:** ^1^ Department of Cardiology, Cardiovascular Diseases Research Centre, School of Medicine Birjand University of Medical Sciences Birjand Iran; ^2^ Student Research Committee Birjand University of Medical Sciences Birjand Iran; ^3^ Infectious Diseases Research Center Birjand University of Medical Sciences Birjand Iran; ^4^ Metabolic Syndrome Research Center Mashhad University of Medical Sciences Mashhad Iran; ^5^ Cellular and Molecular Research Center Birjand University of Medical Sciences Birjand Iran; ^6^ Division of Medical Education Brighton & Sussex Medical School Brighton Sussex UK; ^7^ Clinical Research Development Unit of Akbar Hospital, Faculty of Medicine Mashhad University of Medical Sciences Mashhad Iran; ^8^ Clinical Research Development Unit, Imam Reza Hospital, Faculty of Medicine Mashhad University of Medical Sciences Mashhad Iran

**Keywords:** ferritin, hsCRP, neutrophil, TIBC

## Abstract

Premenstrual syndrome (PMS) and primary dysmenorrhea are common gynecological problems and inflammation may have a role in their etiology. Curcumin is a polyphenolic natural product for which there is increasing evidence of anti‐inflammatory and iron chelation effects. This study assessed the effects of curcumin on inflammatory biomarkers and iron profile in young women with PMS and dysmenorrhea. A sample of 76 patients was included in this triple‐blind, placebo‐controlled clinical trial. Participants were randomly allocated to curcumin (*n* = 38) and control groups (*n* = 38). Each participant received one capsule (500 mg of curcuminoid+ piperine, or placebo) daily, from 7 days before until 3 days after menstruation for three consecutive menstrual cycles. Serum iron, ferritin, total iron‐binding capacity (TIBC) and high‐sensitivity C‐reactive protein (hsCRP), as well as white blood cell, lymphocyte, neutrophil, platelet counts, mean platelet volume (MPV) and red blood cell distribution width (RDW), were quantified. Neutrophil: lymphocyte ratio (NLR), platelet: lymphocyte ratio (PLR), and RDW: platelet ratio (RPR) were also calculated. Curcumin significantly decreased the median (interquartile range) serum levels of hsCRP [from 0.30 mg/L (0.0–1.10) to 0.20 mg/L (0.0–1.3); *p* = 0.041] compared with placebo, but did not show any difference for neutrophil, RDW, MPV, NLR, PLR and RPR values (*p* > 0.05). The treatment schedule was well‐tolerated, and none of markers of iron metabolism statistically changed after the intervention in the curcumin group (*p* > 0.05). Curcumin supplementation may have positive effects on serum hsCRP, a marker of inflammation, with no any changes on iron homeostasis in healthy women with PMS and dysmenorrhea.

## Graphical abstract

Curcumin supplementation significantly decreased serum levels of high‐sensitivity C‐reactive protein (hsCRP) in women with premenstrual syndrome and dysmenorrhea, but could not affect hematological inflammatory parameters including white blood cells (WBC) count, neutrophil percentage, mean platelet volume (MPV) and red blood cell distribution width (RDW). Curcumin was well‐tolerated, and none of markers of iron metabolism statistically changed after intervention.

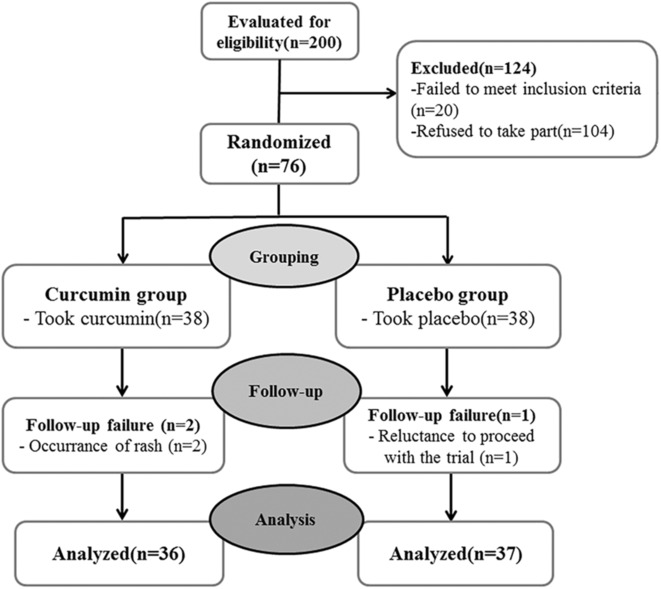

## 1 BACKGROUND

Premenstrual syndrome (PMS) and dysmenorrhea are common menstrual associated complications which have a negative effect on well‐being and quality of life of women of child bearing age (Bahrami, Avan, et al., 2018). PMS is a wide range of cyclically somatic and psychologic symptoms in the late luteal phase which alleviate with menstruation (Bahrami, Bahrami‐Taghanaki, et al., 2018; Bahrami, Gonoodi, et al., 2019). The pain associated with dysmenorrhea is often characterized by the presence of spasmodic‐like pain in the lower abdomen, without any pelvic pathology, before or during of menstrual bleeding (Bahrami et al., 2017). It has been suggested that chronic low grade inflammation as a result of the inflammatory response may have role in the pathophysiology, manifestations and severity of PMS and dysmenorrhea (Barcikowska et al., 2020; Granda et al., 2021).

There are several parameters that can be used to assess hematological biomarkers derived from a complete blood count (CBC), including absolute counts of white blood cells (WBC), neutrophils as well as red blood cell distribution width (RDW), mean platelet volume (MPV), neutrophil: lymphocyte ratio (NLR), platelet: lymphocyte ratio (PLR), and RDW: platelet ratio (RPR) are novel inexpensive and reproducible indices indicating systemic inflammation in different conditions (Bilgin et al., 2020).

RDW estimates erythrocytic heterogeneity in volume. Increased RDW reflects ineffective erythropoiesis and associated with higher cytokine levels such as interleukin (IL)‐1, IL‐6 and tumor necrosis factor‐α (TNF‐α) (He et al., 2018). Lymphocytes and platelet contributed to innate and adaptive immune response(Tomczyńska, & Saluk‐Bijak, 2018). MPV represent platelet activation and indicated inflammation, disease intensity and efficacy of anti‐inflammatory agent in chronic inflammatory diseases.

HsCRP as an acute‐phase reactant released by the liver is strong index of inflammation. It has been shown that serum hsCRP levels vary during the menstrual cycle in PMS women together with physical and mood manifestations, which advocates the contribution of low‐grade inflammation in the etiology of PMS (Puder et al., 2006). Several studies have reported that serum hsCRP, WBC, MPV and NLR are significantly associated with the presence of PMS and dysmenorrhea (Bahrami, Bahrami‐Taghanaki, et al., 2020; Bodur et al., 2017).

Conventional treatment for PMS and dysmenorrhea may induce severe adverse events or be ineffective for relieving pain (Drevon, 1992; Park et al., 2012). Recently, the therapeutic potential of traditional medicine as medicinal herb has attracted considerable attention in relief of menstrual associated pains. Curcumin (CUR) is polyphenolic pigment of perennial herb turmeric (*curcuma longa*) which has pleiotropic effect on multiple molecular targets such as anti‐inflammatory, immunomodulatory and natural antioxidant properties (Bahrami, Sathyapalan, et al., 2020; Parsamanesh et al., 2018). CUR affects the inflammatory response by down‐regulating the expression of inflammatory biomolecules and inhibiting the proinflammatory cytokines release (Bahrami, Majeed, & Sahebkar, 2019; Bahrami, Sadeghnia, et al., 2018). There are several evidences indicated the potential therapeutic effect of CUR on dysmenorrhea pain and PMS severity (Fanaei et al., 2015; Khayat et al., 2015; Kheirkhah, 2016; Rahman et al., 2020).

CUR has been reported to be safe with no serious adverse events even at doses as high as 8–12 g daily for 3 months (Karandish et al., 2021). However, there is evidence that CUR may have iron‐binding capacity, iron chelation and induction of iron metabolism features which leads to iron deficiency anemia (Chin et al., 2014; Jiao et al., 2009), especially in individuals with suboptimal iron concentrations (Shehzad et al., 2017).

Until now, some clinical trials have explored the effects of CUR administration on serum hsCRP value, but to the best of our knowledge have not assessed its effects on hematological parameters and iron metabolism. Taking into account wide‐ranging positive pharmacological and biological characteristics of CUR while we lack controlled trials among patients with both PMS and dysmenorrhea, the aim of this study was to assess the effects of CUR supplementation on iron profile, inflammatory hematological biomarkers and hs‐CRP levels among apparent healthy young women with PMS and dysmenorrhea.

## 2 MATERIALS AND METHODS

### 2.1 Study design and participants

From December 2019 to March 2020, female students residing at five women’ university dormitories in Birjand (South Khorasan province, Iran), were invited for interview in the current randomized, triple‐blind, placebo‐controlled clinical trial. This study was supported by Birjand University of Medical Sciences, and registered at Iranian Registry of Clinical Trial (IRCT20191112045424N1; available at: https://www.irct.ir). This work was part of a comprehensive study, the sample size was calculated for all parameters and the maximum sample size was considered. According to the previous study (Panahi et al., 2016), the sample size was calculated at a power of 80%, *α* = 0.05 and the expectations value of 2.1 mg/L as the difference in mean (d) of serum hsCRP as the primary outcome variable of the study at the end of the trial to reject the null hypothesis. A minimum of 31 patients and final sample size, assuming 20% drop‐out rate was set as 38 patients in each group to give us appropriate power to find an effect compared to the placebo. All participants provided written informed consent for participation. The study protocol was approved by the ethics committee of Birjand University of Medical Sciences and trial was conducted in accord with the Declaration of Helsinki.

Volunteers who fulfilled the following inclusion criteria were recruited into the study: (1) an age between 18 to 24 years; (2) a marital status of single; (3) menstrual cycle days between 21 to 35 days, with the bleeding time lasting 3–7 days; (4) not suffering from gynecological disorders and allergy to herbs or medicinal plants, and (5) definitely diagnosed with both moderate to severe dysmenorrhea and PMS by a gynecologist. Exclusion criteria included participants who: had either acute or chronic disorders based on history and medical examinations, taking any kinds of medications or supplements, and undergone stressful conditions throughout the trial.

Menstrual patterns were evaluated using a questionnaire consisted of items concerning the age of menarche, menstruation cycle days and duration of bleeding. Gynecologist diagnosed women who suffered from dysmenorrhea and PMS by using visual analogue scale (VAS) and PSST (Premenstrual Syndrome Screening Tool), respectively (Ayadilord et al., 2020). According to VAS instrument which rates the dysmenorrhea pain from 0 to 10 (showing no pain to maximum pain; Crichton, 2001), individuals with high menstrual cramps (pain scores ≥8) were considered as dysmenorrhea woman (Osayande & Mehulic, 2014). PMS status and severity was assessed by the PSST questionnaire which is composed of 19 physical and physiological manifestations related to PMS with a four‐point Likert score (0 to 3) which provides a total score varying from 0 to 57 (Siahbazi et al., 2011). Individuals who obtain scores of ≥20 from PSST were considered as PMS female. Finally, young women who experienced moderate to severe dysmenorrhea pain and PMS (VAS score ≥8 and PSST ≥ 20) were enrolled.

**FIGURE 1:**
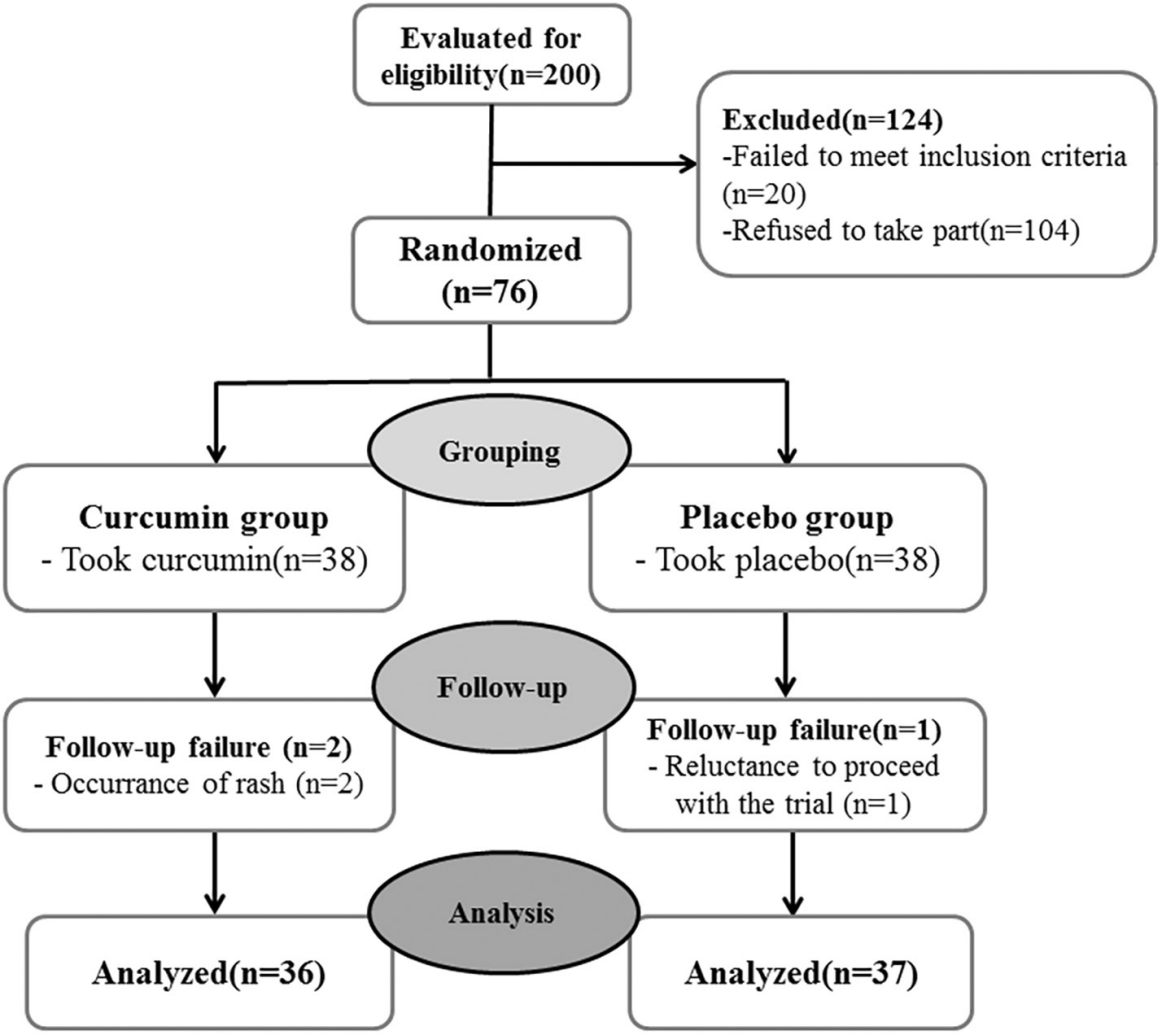
Flow chart of trial.

Although 200 women were eligible to take part in this study, only 76 met the inclusion criteria and were chosen for this trial (Figure 1).

### 2.2 Randomization, blinding and intervention

Eligible participants were randomly divided into either placebo or treatment (CUR) groups. While the CUR consisted of 500 mg curcuminoids +5 mg piperine (95%); C3 Complex (Sami Labs Ltd.), the placebo capsules contained inert filler (500 mg lactose powder) plus 5 mg piperine (Bioperine®, Sami Labs Ltd.). Bioperine® as bioactive alkaloid is purified extract obtained from *Piper species* (black pepper or long pepper) and includes at least 95% piperine, which is an extensively known dietary absorption enhancer. C3 Complex® that was used in the present study contained three major curcuminoids including CUR, demethoxycurcumin and bisdemethoxycurcumin in a patented ratio in single preparation. Previously, the purity of the three major curcuminoids and piperine was determined by HPLC method (Khonche et al., 2016).

With the intent of ensuring the triple‐blind design, CUR and placebo capsules were matched in shape, size and color, and the color of placebo (microcrystalline cellulose) was matched to that of CUR powder. The CUR and placebo powders (labeled as “code A" or “code B") were dispensed in identical blinded bottles. The Pharmacy Department of the Birjand University of Medical Science performed the randomization and blinding. The eligible volunteers were randomly assigned to one of the two arms “code A or B”. Next, a statistician provided a randomized list employing NCSS (statistical software) via the simple block randomization method based on CONSORT guidelines. The eligible study participants were assigned to one of the two groups “code A or B”, regarding to the randomized list. Participants with an even number on the registration list were enrolled to the CUR group; the remaining participants were put into the placebo group. Coding keys were sent to study's principle investigator through mail after the end of study and final analysis. Thus, neither the participants, nor the principal investigator and data analyst had been aware of the group allocation of the participants and had been blind to the capsule ingredients until the trial and data investigation were accomplished. With the aim of improving CUR's oral bioavailability and facilitating its absorption through intestines, piperine, a major bio‐active component of pepper was used in addition to CUR (Shoba et al., 1998). The study subjects followed a regimen of a capsule per day for the duration of 10 days (7 days prior and 3 days subsequent to the onset of the menstrual bleeding) for 3 consecutive cycles, based on previous study (Khayat et al., 2015). Degree of adherence, participants' well‐being, and drug tolerability were assessed within and after the trial per week in both groups by telephone follow‐up. Moreover, capsule adherence was evaluated by asking subjects to provide an estimate of the consistency of capsule intake per cycle and a count of returned capsules at month 3 was also performed. Participants were counseled to retain their routine regimen and physical activities and avoided consuming any additional supplements.

### 2.3 Blood collection

Twenty milliliter blood specimens were collected in the morning subsequent to a 12‐h overnight fasting. Blood samples were obtained from each participant 3 days prior to the intervention and within 3 days subsequent to the consuming last capsule. We used both serum separator tubes and EDTA tubes for the purpose of this study. Serum samples were stored −80°C in a reference laboratory until analysis. EDTA tube was used to quantify blood cell counts.

### 2.4 Complete blood count

Blood cell counts, hemoglobin levels, dimensional variables (MPV and RDW), and some combined parameters, such as NLR, PLR and RPR, were assessed by means of an automated commercial cell counter (Sysmex K‐800). All experiments were conducted in duplicate.

### 2.5 Measurement of hsCRP and iron profile

Serum hsCRP, Fe and total iron‐binding capacity (TIBC) levels were evaluated using commercial kits (Pars Azmun, Iran; catalogue numbers: 1027015, 1,022,001, 1,022,032 respectively) with auto‐analyzer (Prestige 24i, Tokyo Boeki Ltd.). Serum ferritin values were quantified using an enzyme‐linked immunosorbent assay (ELISA kit; Diazist, Tehran, Iran; RRID:AB_10575316) according to the manufacturer's instructions. Each test was performed in duplicate.

### 2.6 Other variables

The anthropometric parameters and blood pressure of participants were measured using standard procedures in our health centers by expert paramedic staff as described previously (Askari et al., 2020).

The dietary intake of study population was evaluated by a trained dietician by using a three‐day recall food records method (two weekdays and one weekend day) at the first and last week of the trial. Dietplan software (version 4, Forestfield software Ltd.) was used for estimation of daily mean of energy and macro‐ and micronutrients intake during the trial.

### 2.7 Statistical analysis

Statistical data analyses were performed by using the Statistical Package for the Social Science software for Windows, version 18.0 (SPSS Inc.). All indices were presented as mean ± standard deviation (SD) or median and interquartile range (IQR). Non‐parametric tests were also used in the statistical analysis due to some parameters were not normally distributed (Kolmogorov–Smirnov test). The Student's *t*‐test (for non‐normally distributed parameters) or Mann–Whitney U test (for non‐normally distributed parameters) was recruited to identify any differences between the two CUR and placebo groups. For normally and non‐normally variables, paired sample *t*‐test and Wilcoxon signed‐rank test were used for comparing between pre‐and‐post variables within groups, respectively. Analysis of covariance (ANCOVA) was employed to find any significant differences between the two groups at the end of the trial, adjusting for baseline values and known confounding variables (changes of BMI and score of PSST and VAS throughout the study). *p* values ≤0.05 were set to illustrate statistical significance.

## 3 RESULTS

### 3.1 Recruitment

A total of 76 subjects were recruited into the trial and 38 individuals were allocated to each of the groups. Of the total, 73 subjects completed the study: 36 in CUR group and 37 in placebo group. During the trial, 2 patients who reported rash side effects in the CUR group and one participant in the placebo group who unwilling to continue the study were lost to follow‐up (Figure 1). There was no significant difference in the drop‐out rate between the two parallel arms (*p* = 0.07).

### 3.2 Comparison of baseline features between the two groups

Comparison of the baseline features of participants indicated no significant differences between CUR and placebo groups regarding to the mean age, blood pressure and anthropometric indices (Table 1). In addition, dietary intakes of participants including mean energy, macro‐ and micro‐nutrient intake, showed no significant differences between the two arms before and after trial (*p* > 0.05; Table 2).

**TABLE 1:** Baseline features of study population.

Variable	Curcumin (*n* = 36)	Placebo (*n* = 37)	*p* value*
Age (year)	21.1 ± 1.8	21.0 ± 1.4	0.87
WC (cm)	70.8 ± 5.7	70.4 ± 6.8	0.75
Weight (kg)	54.7 ± 8.2	54.6 ± 7.8	0.96
Height (cm)	161.3 ± 5.6	162.5 ± 4.9	0.17
BMI (kg/m^2^)	21.4 ± 2.2	21.0 ± 3.4	0.54
SBP (mmHg)	106.3 ± 97.4	106.4 ± 96.3	0.95
DBP (mmHg)	70.8 ± 8.1	70.5 ± 7.2	0.82

*Note*: Data presented as mean ± SD.

Abbreviations: BMI, body mass index; DBP, diastolic blood pressure; SBP, systolic blood pressure; WC, Waist circumference.

* Using independent sample *t*‐test.

**TABLE 2:** Dietary intakes of the study groups at baseline and after intervention.

Variables	Curcumin (*n* = 36)	Placebo (*n* = 37)	*p* value^a^
Total energy (kcal)			
Before	2169 ± 719	2193 ± 780	0.63
After	2120 ± 697	2098 ± 770	0.92
*p* value^b^	0.82	0.98	
Carbohydrate (g/d)			
Before	62.31 ± 30.9	67.9 ± 36.7	0.22
After	61.39 ± 22.86	62.4 ± 31.6	0.87
*p* value^b^	0.69	0.71	
Protein (g/d)			
Before	31.83 ± 13.2	33.73 ± 14.80	0.32
After	32.71 ± 12.5	33.61 ± 14.31	0.81
*p* value^b^	0.83	0.92	
Dietary fiber (g/d)			
Before	6.78 ± 3.05	7.31 ± 2.93	0.69
After	7.23 ± 2.52	7.23 ± 3.46	0.88
*p* value^b^	0.55	0.94	
Total fat (g/d)			
Before	12.89 ± 9.77	14.81 ± 7.92	0.46
After	14.74 ± 10.23	15.66 ± 9.44	0.64
*p* value^b^	0.27	0.83	
Vitamin C (mg/d)			
Before	120.2 ± 129.8	109.1 ± 91.3	0.72
After	138.0 ± 124.3	124.2 ± 94.5	0.43
*p* value^b^	0.77	0.81	
Vitamin A (RE)			
Before	41.00 ± 38.13	53.12 ± 56.32	0.25
After	40.9 ± 40.92	54.01 ± 48.86	0.17
*p* value^b^	0.71	0.92	
Thiamin (mg)			
Before	0.35 ± 0.22	0.40 ± 0.18	0.70
After	0.43 ± 0.29	0.38 ± 0.30	0.76
*p* value^b^	0.34	0.77	
Niacin (mg)			
Before	7.92 ± 2.43	6.20 ± 4.53	0.20
After	8.33 ± 3.09	6.41 ± 2.92	0.08
*p* value^b^	0.83	0.60	
Calcium (mg)			
Before	208.3 ± 114.0	240.2 ± 141.7	0.21
After	221.3 ± 101.6	246.1 ± 162.3	0.53
*p* value^b^	0.59	0.85	
Phosphorus (mg)			
Before	417.2 ± 131.2	477.8 ± 144.2	0.12
After	442.9 ± 121.7	451.1 ± 162.3	0.78
*p* value^b^	0.64	0.74	
Magnesium (mg)			
Before	100.2 ± 38.2	99.3 ± 37.6	0.83
After	123.1 ± 23.0	97.1 ± 41.6	0.21
*p* value^b^	0.74	0.80	
Manganese (mg)			
Before	1.34 ± 0.55	1.60 ± 0.71	0.61
After	1.53 ± 0.91	1.50 ± 0.72	0.71
*p* value^b^	0.37	0.33	
Zinc (mg/d)			
Before	2.51 ± 0.83	2.53 ± 1.11	0.87
After	2.63 ± 0.92	2.83 ± 1.22	0.82
*p* value^b^	0.96	0.74	

*Note*: Data presented as mean ± SD and adjusted for energy intake.

^a^ Independent sample *t*‐test.

^b^ Paired sample *t*‐test.

### 3.3 Effect of the intervention on inflammatory biomarkers

**FIGURE 2:**
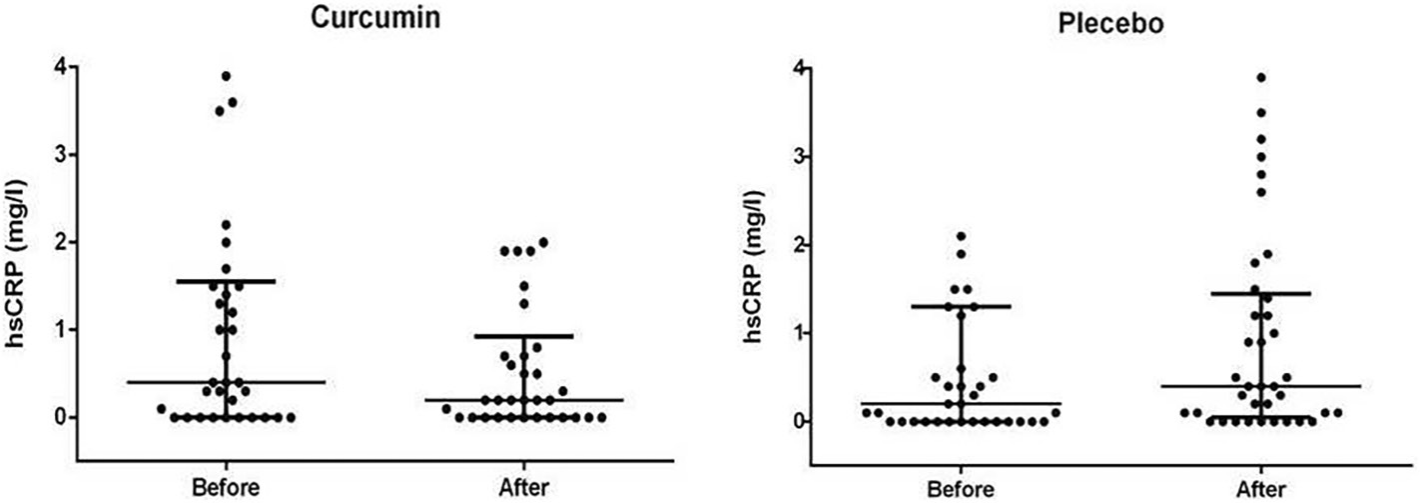
Comparison of hsCRP levels in curcumin and placebo groups before and after intervention.

After three menstrual cycle of intervention, significant decrement was observed in the median (IQR) serum levels of hsCRP in the CUR group [from 0.30 mg/L (0.0–1.10) to 0.20 mg/L (0.0–1.3); *p* = 0.039], but no significant changes were detected in the placebo group [from 0.10 mg/L (0.0–0.6) to 0.40 mg/L (0.0–1.5); *p* = 0.38; Figure 2]. However, within‐group analysis of hematological inflammatory parameters including WBC count, neutrophil percentage, RDW, MPV, NLR, PLR and RPR did not reach statistical significant difference in the both CUR and placebo treated group (*p* > 0.05; Table 3). At the end of the study, between‐group analysis by ANCOVA showed only significant differences in hsCRP index (*p* = 0.041).

**TABLE 3:** Comparison of inflammatory biomarkers in treatment groups before and after intervention.

Variables	Measurement period	Curcumin	Placebo	*p* value^a^
WBC (10^9^ cells/L)	Before intervention	6.6 ± 1.9	6.6 ± 1.4	0.83
	After intervention	7.1 ± 1.8	6.7 ± 1.8	0.39
	*p* value^b^	0.13	0.83	
Neutrophil (%)	Before intervention	49.2 ± 7.6	52.1 ± 7.8	0.37
	After intervention	47.1 ± 9.8	48.0 ± 8.2	0.93
	*p* value^b^	0.45	0.31	
RDW (%)	Before intervention	13.2 ± 1.6	12.8 ± 0.4	0.09
	After intervention	13.4 ± 1.5	12.9 ± 0.4	0.15
	*p* value^b^	0.21	0.84	
MPV (fL)	Before intervention	10.2 ± 0.74	10.1 ± 1.0	0.21
	After intervention	10.2 ± 0.69	10.3 ± 1.2	0.53
	*p* value^b^	0.49	0.33	
NLR	Before intervention	1.2 ± 0.41	1.6 ± 0.62	0.23
	After intervention	1.2 ± 0.56	1.3 ± 0.5	0.76
	*p* value^b^	0.91	0.17	
PLR	Before intervention	6.7 ± 1.8	7.7 ± 2.4	0.07
	After intervention	6.7 ± 2.2	6.9 ± 3.1	0.81
	*p* value^b^	0.94	0.28	
RPR	Before intervention	0.049 ± 0.01	0.48 ± 0.01	0.82
	After intervention	0.050 ± 0.01	0.054 ± 0.02	0.33
	*p* value^b^	0.22	0.13	
hsCRP (mg/L)	Before intervention	0.30 (0.0–1.10)	0.10 (0.0–0.6)	0.65
	After intervention	0.20 (0.0–1.3)	0.40 (0.0–1.5)	**0.041**
	*p* value^b^	**0.039**	0.38	

Bold values significant *p* < 0.05.

*Note*: Values expressed as mean ± SD (normally distributed variables) or median and interquartile range (non‐normally distributed variables).

Abbreviations: hsCRP, high‐sensitivity C‐reactive protein; MPV, mean platelet volume; NLR, neutrophil: lymphocyte ratio; PLR, platelet: lymphocyte ratio; RDW, red blood cell distribution width; RPR, red blood cell distribution width: platelet ratio; WBC, White blood cell.

^a^ *p* values indicate comparison between groups by using independent sample *t*‐test (normally distributed variables) or Mann–Whitney (non‐normally distributed variables) at baseline and ANCOVA test after treatment.

^b^ *p* values indicate comparison within groups by using paired‐sample *T* test (normally distributed variables) or Wilcoxon signed‐rank test (non‐normally distributed variables).

### 3.4 Effect of the intervention on iron profile

The blood levels of Fe, ferritin, TIBC and hemoglobin showed no significant difference between the two groups at the baseline (*p* > 0.05; Table 4). Moreover, the paired sample *t*‐test and Wilcoxon signed‐rank test revealed no significant difference at the end of the trial regarding to the mean iron profile values in the studied groups (*p* > 0.05).

**TABLE 4:** Comparison of iron profile in treatment groups before and after intervention.

Variables	Measurement period	Curcumin group	Placebo group	*p* value^a^
Fe (μg/dL)	Before intervention	74.5 ± 44.8	87.6 ± 45.0	0.22
	After intervention	67.4 ± 33.0	78.7 ± 36.0	0.25
	*p* value^b^	0.37	0.37	
TIBC (μmol/L)	Before intervention	118.6 ± 68.8	137.4 ± 77.6	0.30
	After intervention	132.7 ± 56.2	113.9 ± 65.5	0.20
	*p* value^b^	0.37	0.16	
Ferritin (ng/dL)	Before intervention	11.4 (10.1–23.5)	18.6 (12.8–61.5)	0.088
	After intervention	10.5 (9.9–52.4)	10.7 (9.3–37.7)	0.71
	*p* value^b^	0.62	0.071	
Hb (g/dL)	Before intervention	13.7 ± 1.4	14.3 ± 0.9	0.63
	After intervention	13.6 ± 1.3	14.4 ± 0.6	0.17
	*p* value^b^	0.98	0.75	

*Note*: Values expressed as mean ± SD (normally distributed variables) or median and interquartile range (non‐normally distributed variables).

Abbreviations: Hb, hemoglobin; hsCRP, High‐sensitivity C‐reactive protein; TIBC, total iron‐binding capacity.

^a^ *p* values indicate comparison between groups by using independent sample *t*‐test (normally distributed variables) or Mann–Whitney (non‐normally distributed variables) at baseline, and ANCOVA test after treatment.

^b^ *p* values indicate comparison within groups by using paired‐sample *T* test (normally distributed variables) or Wilcoxon signed‐rank test (non‐normally distributed variables).

## 4 DISCUSSION

To the best of our knowledge, this study is the first clinical trial that has investigated the effects of CUR intervention on inflammatory markers and iron profile in women with both PMS and dysmenorrhea. Our study demonstrated that 3 successive menstrual cycle administration of oral CUR had considerable effect on hsCRP levels which decreased significantly in the CUR group by within‐groups analyses. CUR had no significant effect on measures of iron homeostasis in healthy women with PMS and dysmenorrhea.

In our study, CUR oral supplements significantly reduced serum hsCRP levels. The results of two randomized clinical trial disclosed that nano‐formulate of CUR significantly mitigated mean serum hsCRP levels in patients with nonalcoholic fatty liver disease (Jazayeri‐Tehrani et al., 2019), and hemodialysis patients (Vafadar_Afshar et al., 2020). However, serum hsCRP concentrations was not alter after CUR treatment in overweight/obese women with polycystic ovary syndrome (Sohaei et al., 2019), and patients with diabetic foot ulcer (Mokhtari et al., 2021). Notably, serum hsCRP levels were significantly decreased after the trial in both the CUR and placebo groups in patients with chronic cutaneous complications. Though, the net change of decrement was significantly higher in the CUR group (Panahi et al., 2012). In two recent meta‐analysis of randomized controlled trials presented, CUR significantly decreased CRP and hsCRP levels (Ferguson et al., 2020; Gorabi et al., 2021). It has been suggested that CUR intervention may reduce the serum levels of hsCRP, dependent on the bioavailability of CUR formulations, dose and duration of supplementation (Sahebkar, 2014).

The anti‐inflammatory effects of CUR might be associated with its phytochemical compounds. The potential positive effect of CUR on inflammatory response may be because of the modulatory effect of CUR on inflammatory signaling paths. It prohibits myeloid differentiation protein 2‐Toll‐like receptor 4 co‐receptor axes, induces peroxisome proliferator activated receptor‐γ (PPAR‐γ), suppresses the induction and phosphorylation of JAK/STAT proteins, and blocks mitogen activated protein kinase (MAPKs) cascade (Aggarwal et al., 2003; Kocaadam & Şanlier, 2017). CUR also inhibits infiltration, stimulation, maturation, rolling and adhesion of leukocytes, as well as the generation of proinflammatory molecules at the inflammation site. Curcumin was reported to suppress NF‐kB, decreased secretion, expression and release of proinflammatory cytokines, such as IL‐6, IL‐1β, TNF‐α, and then eventually reduced hsCRP generation in hepatocyte (Tabrizi et al., 2019).

Since hs‐CRP has a non‐normal distribution, most studies present it as median (25th, 75th percentile) or apply log transformation or divide it into quartiles or quintiles for comparison (Rogowski et al., 2004; Yang et al., 2018). We found median levels of hs‐CRP significantly decreased in patients receiving curcumin from 0.30 [IQR: 0.0–1.10] mg/L to 0.20[IQR: 0.0–1.3] mg/L using the Wilcoxon test, which compares matched‐pair data, based on differences with a variable with a non‐normal distribution, such is the case for hs‐CRP.

CUR had no significant effect on inflammatory hemogram parameters in this trial. Results of recent randomized clinical trial on critically ill patients with sepsis failed to demonstrate the nano‐curcumin had beneficial effect on NLR and PLR compared to placebo (Naeini et al., 2022). Consistently, CUR (1500 mg/day) intake for 8 weeks could not significantly affect RDW, WBC and NLR in patients with ulcerative colitis (Sadeghi et al., 2020). It has been shown that the anti‐inflammatory effect of CUR is increased with longer duration of treatment in which, the utmost effect was observed nearly after 13 weeks of intervention (Ferguson et al., 2020; White et al., 2019). In the present study, the relatively short follow‐up and single dose of treatment may have restricted the effect size of CUR and so is the reason for the absence of observed efficacy regarding to the improving hematological biomarkers.

Our results indicate that three menstrual cycle CUR administration did not alter iron status in healthy women with PMS and dysmenorrhea. We evaluated iron status by measuring serum iron, ferritin and TIBC levels in our cases because decreased iron status was reported post long‐time curcuminoid feeding in previous in vivo studies (Chin et al., 2014; Jiao et al., 2009). Two previous studies among β‐thalassemia major patients and moderately hyperlipidemic subjects reported that CUR was unable to decrease hemoglobin, serum iron and ferritin concentrations significantly (Kocher et al., 2016; Nasseri et al., 2017). But, Mohammadi et al. reported that 12 weeks CUR treatment did not significantly alter hemoglobin, transferrin, TIBC and ferritin levels in β‐thalassemia patients; nonetheless, a considerable reduction was found in ferritin concentrations (*p* = 0.059). In this latter study, when subjects in the CUR group were subgrouped according to their baseline serum ferritin levels, the effects of CUR supplementation was found to be statistically significant only for the subgroup with serum ferritin levels >1500 ng/mL (*p* = 0.04), indicating that CUR could decrease ferritin in patients with severe iron overload (Mohammadi et al., 2018).

CUR acts as a free radical scavenger because of the enol form being a hydrogen donor or acceptor and has been reported to neutralize the detrimental effects of iron‐induced ROS generation (Ferrari et al., 2014). Curcumin moderately forms chelate iron‐forming insoluble complexes which may affect iron absorption in the gut, though this has largely been reported in in vitro experiments (Ak & Gulcin, 2008). However, CUR possibly has iron‐chelating activity through direct binding of the 𝛽‐diketone moiety to ferric ions and regulating the transcription of proteins implicated in iron depletion like hepcidin and transferrin receptor (Jiao et al., 2009). It seems that the inconsistency in the findings of various studies is due to the different study design and clinical features of the cases, case‐by‐case variability in absorption of CUR and doses, formulation and duration of the administration.

This was a sub‐study of our previous triple‐blinded controlled trial on CUR's effects on menstrual‐associated manifestations in young women with PMS and dysmenorrhea (Bahrami et al., 2021). There are several limitations of this study. First, we only included young single women, so our result was not generalized to all women population. Second, it is possible that three menstrual cycle supplementation of CUR there is not adequate time to affect the inflammatory factors involved in this trial. Short duration of intervention also prevent judgment on the long‐term efficacy of CUR intake especially in terms of iron metabolism in young women. Future trials with larger scale using higher doses and duration as well as measurement of bioavailability and blood CUR levels and quantification of additional inflammatory biomarkers are warranted to support our findings.

## 5 CONCLUSION

In conclusion, CUR was significantly more effective versus placebo in decreasing serum hsCRP, an inflammatory biomarker, in young women with PMS and dysmenorrhea. There are no significant differences between CUR intake and placebo regarding to the iron profile, while no treatment‐induce iron deficiency anemia were registered. However, further studies are warranted to get a definitive conclusion.

## AUTHOR CONTRIBUTIONS

Afsane Bahrami conducted all analyses and drafted the manuscript. Amir Talebpour and Mahtab Mohammadifard coordinated the fieldwork of the study. Reza Zare Feyzabadi, Mahboube Tajik, Sara Mahmoudzadeh, Mansoore Saharkhiz and Hadis Rezapour provided methodological feedback. Afsane Bahrami and Gordon A. Ferns supervised the overall research project and helped to draft the manuscript. All of the authors have read and confirmed the final manuscript. All authors state that they have no conflicts of interest.

## FUNDING INFORMATION

This study was supported by grants [grant nu#456131(Afsane Bahrami)] from Birjand University of Medical Sciences, Birjand, Iran.

## CONFLICT OF INTEREST STATEMENT

The authors have no conflict of interest to disclose.

## CONSENT TO PUBLISH

Not applicable.

## ETHICS APPROVAL

Ethical approval was obtained from the Birjand University of Medical Sciences and informed written consent was completed by all participants (code: IR.BUMS.REC.1399.226).

## INFORMED CONSENT

Informed consent was obtained from all individual participants included in the study.

## TRIAL REGISTRATION

Iranian Registry of Clinical Trial (registered prospectively: IRCT20191112045424N1; available at: https://www.irct.ir/trial/43634).

## Data Availability

The datasets used and analyzed during the current study are available from the corresponding author on reasonable request.
